# Influence of slip on the Plateau–Rayleigh instability on a fibre

**DOI:** 10.1038/ncomms8409

**Published:** 2015-06-12

**Authors:** Sabrina Haefner, Michael Benzaquen, Oliver Bäumchen, Thomas Salez, Robert Peters, Joshua D. McGraw, Karin Jacobs, Elie Raphaël, Kari Dalnoki-Veress

**Affiliations:** 1 Department of Experimental Physics, Saarland University, D-66041 Saarbrücken, Germany; 2 Department of Physics and Astronomy, McMaster University, 1280 Main Street West, Hamilton, Ontario, Canada, L8S 4M1; 3 PCT Lab, UMR CNRS 7083 Gulliver, ESPCI ParisTech, PSL Research University, 75005 Paris, France; 4 Max Planck Institute for Dynamics and Self-Organization (MPIDS), 37077 Göttingen, Germany

## Abstract

The Plateau–Rayleigh instability of a liquid column underlies a variety of fascinating phenomena that can be observed in everyday life. In contrast to the case of a free liquid cylinder, describing the evolution of a liquid layer on a solid fibre requires consideration of the solid–liquid interface. Here we revisit the Plateau–Rayleigh instability of a liquid coating a fibre by varying the hydrodynamic boundary condition at the fibre–liquid interface, from no slip to slip. Although the wavelength is not sensitive to the solid–liquid interface, we find that the growth rate of the undulations strongly depends on the hydrodynamic boundary condition. The experiments are in excellent agreement with a new thin-film theory incorporating slip, thus providing an original, quantitative and robust tool to measure slip lengths.

## Abstract (web summary)

A thin liquid coating on a fibre can break up into droplets due to the Plateau–Rayleigh instability, as for instance on a spider web. Here, Haefner *et al*. show that the growth rate of the droplet undulations strongly depends on the fibre–liquid boundary condition and slip accelerates the instability.

## Graphical abstract

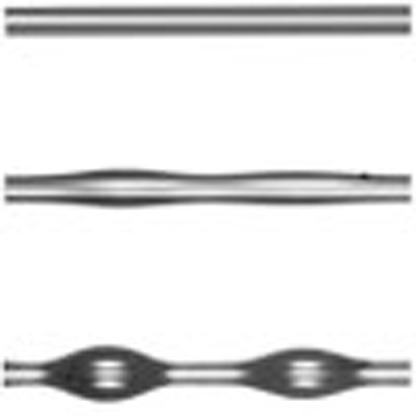

Glistening pearls of water on a spider's web^1^ or the breakup of a cylindrical jet of water into droplets are familiar manifestations of the Plateau–Rayleigh instability (PRI)^2^,^3^. By evolving into droplets, the surface area of the liquid and consequently the surface energy are reduced. This instability also acts for a liquid film coating a solid fibre^4^,^5^,^6^,^7^,^8^,^9^,^10^,^11^,^12^, a situation where the flow boundary condition at the solid–liquid interface provides additional complexity to the system. Although the breakup of a homogeneous film into droplets on a fibre may be a nuisance in coating technologies of, for example, wires and optical fibres, this fundamental instability turns out to be very useful: take for example water collection through fog harvesting^13^,^14^, a biomimetic approach that is perfected in nature by the spider's web^1^. Hence, taking advantage of the PRI on a fibre enables gaining physical insight into the solid–liquid boundary condition. Indeed, understanding the breakdown of the no-slip boundary condition^15^ is of major interest to the scientific and industrial communities, as it has practical implications in areas that involve small-scale fluid systems, such as lab-on-a-chip devices, flows in porous media, as well as biological flows, to name a few.

The classical case of the breakup of a laminar free-flowing liquid stream is dependent on material properties and geometry^16^. Although there are some studies of this instability in geometries other than free cylindrical liquid flows^17^,^18^,^19^,^20^,^21^,^22^,^23^, the role of the boundary condition at the solid–liquid interface on the evolution of the PRI is less well understood^24^,^25^. It has been shown that the appearance of a dewetting liquid rim undergoing a Plateau–Rayleigh-type instability is influenced by the hydrodynamic boundary condition at the solid–liquid interface^26^; however, a general quantitative match between the growth dynamics of the PRI in experiments and analytical theory involving hydrodynamic slip has thus far not been achieved.

Characterization of slip boundary conditions at interfaces and their controlling parameters has been actively investigated in the literature^27^,^28^,^29^,^30^,^31^. The classical boundary condition assumes no slip at the solid–liquid interface. That is, for simple shear flow in the *x* direction along a solid interface placed at *z*=0, the tangential flow velocity, *v*_*x*_(*z*), vanishes at the solid–liquid interface: *v*_*x*_(0)=0. However, as already noted by Navier^32^, there is no fundamental principle requiring *v*_*x*_(0)=0 and one can also have hydrodynamic slippage at this interface, as defined by the slip length: *b*=[*v*_*x*_/∂_*z*_*v*_*x*_]|_*z*=0_, where *b*=0 corresponds to the classical no-slip case.

Here we explicitly address the effect of a varying boundary condition, from no-slip to slip at the solid–liquid interface, on the PRI of a viscous liquid layer on a solid fibre. We find that the growth rate of the instability is strongly affected by the solid–liquid interface, with a faster breakup into droplets for a slip boundary condition compared with an equivalent sample with no slip. In contrast, the wavelength λ* of the fastest growing mode is not sensitive to the solid–liquid interface. The linear stability analysis of a newly developed thin-film equation incorporating slip is in excellent agreement with our data. The theory is valid for all Newtonian liquids and enables the precise determination of the capillary velocity *γ*/*η* and most importantly of the slip length *b*.

## Results

### The experimental approach

**Figure 1:**
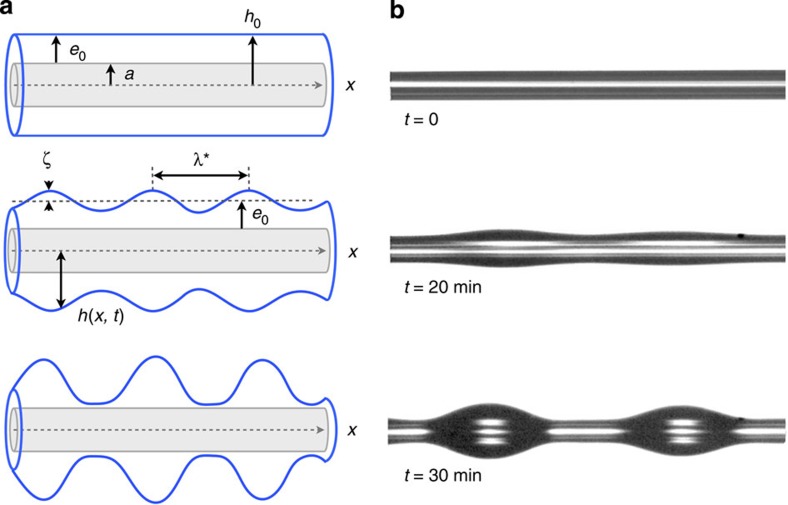
Plateau–Rayleigh instability on a fibre. (**a**) Schematic and (**b**) optical micrographs illustrating the PRI for a liquid PS film on a glass fibre. At *t*=0, the PS film on the no-slip fibre has a thickness *e*_0_=13.2±1 μm and the glass fibre radius is *a*=9.6±1 μm. The width of the optical images is 560 μm.

An entangled polystyrene (PS, 78 kg mol^−1^) film with homogeneous thickness *e*_0_ (5–93 μm) is coated onto a fibre with radius *a* (10–25 μm), resulting in a PS-coated fibre with radius *h*_0_=*a*+*e*_0_, as schematically shown in Fig. 1a. Glass fibres provide a simple no-slip boundary condition^33^. In contrast, a slip interface results from coating the entangled PS film onto a glass fibre pre-coated with a nanometric thin amorphous fluoropolymer (AF2400, 14±1 nm)^34^. The fluoropolymer coating on glass was used, because it is well established that PS, above a critical molecular weight (*M*_c_∼35 kg mol^−1^), exhibits significant hydrodynamic slip at this solid–liquid interface^34^. Henceforth, we will refer to these as the ‘no-slip' and ‘slip' fibres.

All samples were prepared and stored at room temperature, well below the PS glass transition temperature (*T*_g_∼100 °C), thereby ensuring that there is no flow in the PS film before the start of the experiment. Before each experiment, the PS-coated fibre was measured with optical microscopy as shown in the *t*=0 image of Fig. 1b. To initiate the experiments, samples were annealed in ambient atmosphere at 180 °C—well above *T*_g_—which causes the PRI to develop. The evolution of the surface profile was recorded with optical microscopy. Figure 1b shows a typical evolution for a PS film on a no-slip fibre.

Above *T*_g_, the liquid PS film becomes unstable, which causes variations in the local axisymmetric surface profile, *h*(*x*, *t*)=*h*_0_+*ζ*(*x*, *t*), over the axial coordinate *x* and time *t*. The amplitude *ζ*(*x*, *t*) of the undulations grows with time and finally results in a droplet pattern displaying a uniform wavelength. By measuring the spatial variation of *ζ*(*x*, *t*) in the initial development and locating the maxima, the PRI wavelength λ* of each sample was determined (see Fig. 1a). Typically, four or five wavelengths were averaged per sample. In addition, by measuring the temporal change in radius of an individual bulge, we gained information about the growth rate of the instability and consequently the influence of the hydrodynamic boundary condition.

### The theoretical approach

The experimental findings can be understood within the lubrication approximation, from a thin-film model based on the Laplace pressure-driven Stokes equation. We assume incompressible flow of a viscous Newtonian liquid film of thickness varying from 5 to 93 μm, which is well above the film thickness where disjoining pressure plays a role (a few tens of nanometres)^35^. Gravitational effects can be neglected, as all length scales involved in the problem are well below the capillary length *l*_c_≃1.73 mm. Finally, the velocities of the liquid films are small (for example, the fastest observed rate of change in the amplitude is ∼25 nm s^−1^). Thus, the Reynolds and Weissenberg numbers are orders of magnitude smaller than 1, and inertial and viscoelastic effects can be ignored.

We non-dimensionalize the problem (see Fig. 1 for variable definitions) through


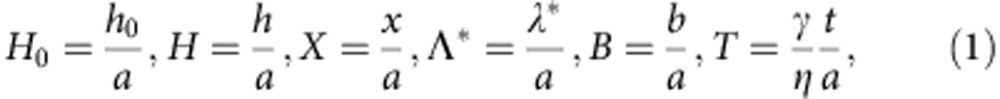


where the capillary velocity *γ*/*η* is the ratio of the liquid–air surface tension to the viscosity of PS. By assuming volume conservation, no stress at the liquid–air interface and the Navier slip condition at the solid–liquid boundary, one obtains (see Supplementary Methods) the governing equation for the dimensionless profile *H*(*X*, *T*)


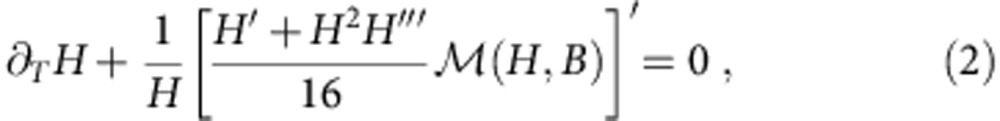


where


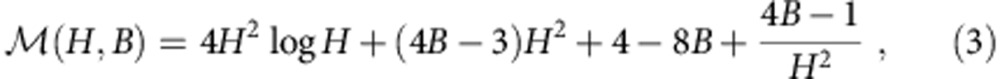


and where the prime denotes the partial derivative with respect to *X*. It is noteworthy that equation (2) is a composite equation in the sense that we have kept a second-order lubrication term in the pressure contribution: the axial curvature. It is the lowest order term counterbalancing the driving radial curvature and it is thus crucial to obtain the actual threshold of the instability. The dynamical aspect of the flow is however well described at the lowest lubrication order. An interesting discussion on this matter can be found in ref. 9.

Performing linear stability analysis, namely letting *H*(*X*, *T*)=*H*_0_+*ɛ*(*T*)*e*^*iQX*^, where *ɛ*(*T*)<<1, yields an exponential growth of the perturbation of the form *ɛ*(*T*) ∝*e*^*T/τ*(*Q*)^, where the rate function is given by


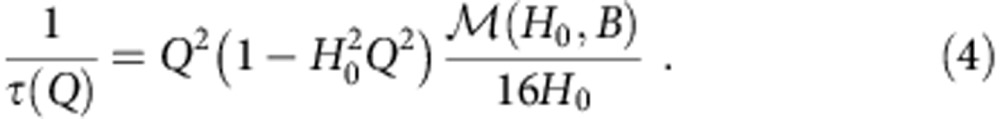


We define the fastest growing mode Q* corresponding to the smallest time constant τ*=*τ*(Q*) and obtain Q*=1/(*H*_0_√2). Writing Q* in terms of the dimensionless wavelength leads to


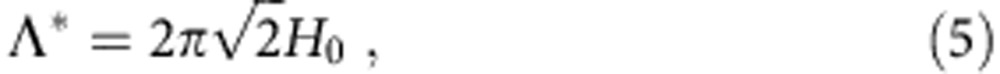


which is similar to the classical PRI dominant wavelength for inviscid jets with no solid core. We note that the correspondence between Rayleigh's result and ours is dependent on both the assumption of Stokes flow and on the presence of a solid core in our system.

The dimensionless growth rate of the fastest growing mode is given by


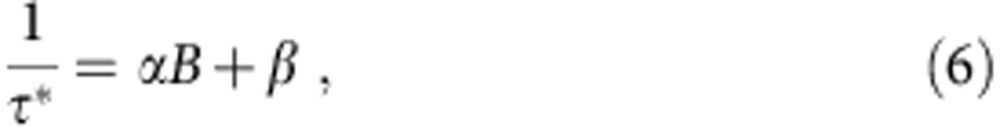


where *B* is the dimensionless slip length, defined in equation (1), and where *α* and *β* are parameters that depend on geometry only


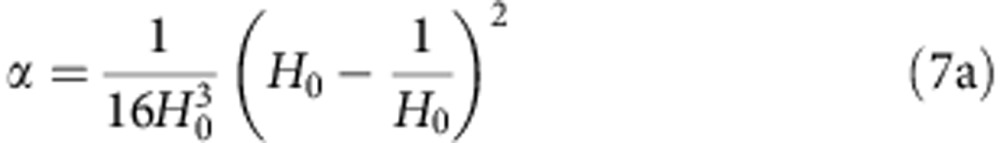



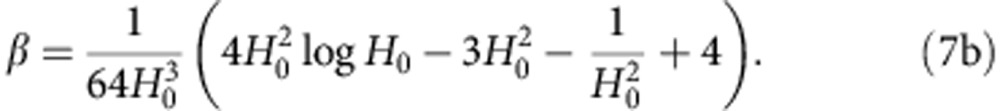


As one sees, the wavelength of the fastest growing mode depends exclusively on the initial total radius, whereas the corresponding growth rate is a linear function of the slip length. It is worth noting that in the case of shear-thickening liquids, the PRI should be significantly slowed down as a result of the increasing viscosity associated with an increasing strain rate. Conversely, a shear-thinning liquid is expected to accelerate the rise of the instability. In the case of a viscoelastic material, Maxwell-like rheological models^36^ can be implemented and may reveal interesting physics beyond the scope of the present study.

### The spatial evolution of the instability

**Figure 2:**
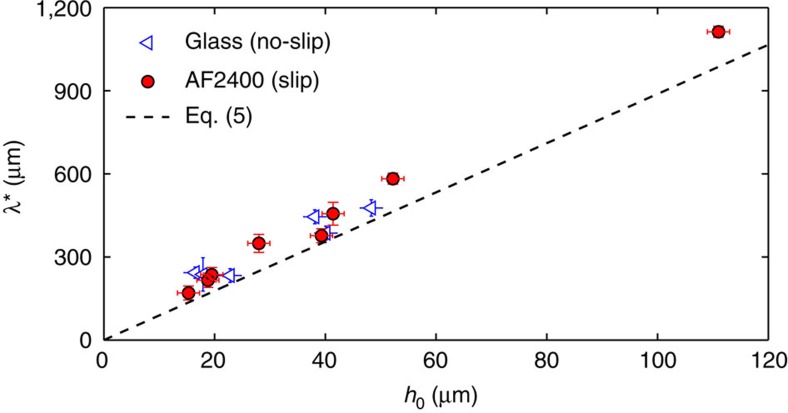
Influence of the geometry on the wavelength of the instability. Wavelength of the fastest growing mode as a function of the total initial radius (see Fig. 1a). The black dashed line represents equation (5). The error bars are calculated from the error in the geometry and the inaccuracy given by the wavelength measurement.

Figure 2 displays the wavelength λ* of the fastest growing modes, measured on no-slip and slip fibres, as a function of the initial total radius *h*_0_. As expected from equation (5), the wavelength λ* grows linearly with increasing radius of the fibre–polymer system and is identical on slip and no-slip fibres. This is consistent with experiments and a theoretical framework for retracting liquid ridges on planar substrates^26^. The spatial morphology of the instability at short times is thus unaffected by the solid–liquid boundary condition. Although it is clear from Fig. 2 that the wavelength is the same on slip and no-slip fibres, there is a small systematic deviation from the theory. This slight deviation could perhaps be related to the lowest lubrication order of the present model^9^, but could also be attributed to the experimental contribution of several modes and the asymmetry of the rate function (see equation (4)) in the vicinity of the fastest growing mode.

### The temporal evolution of the instability

**Figure 3:**
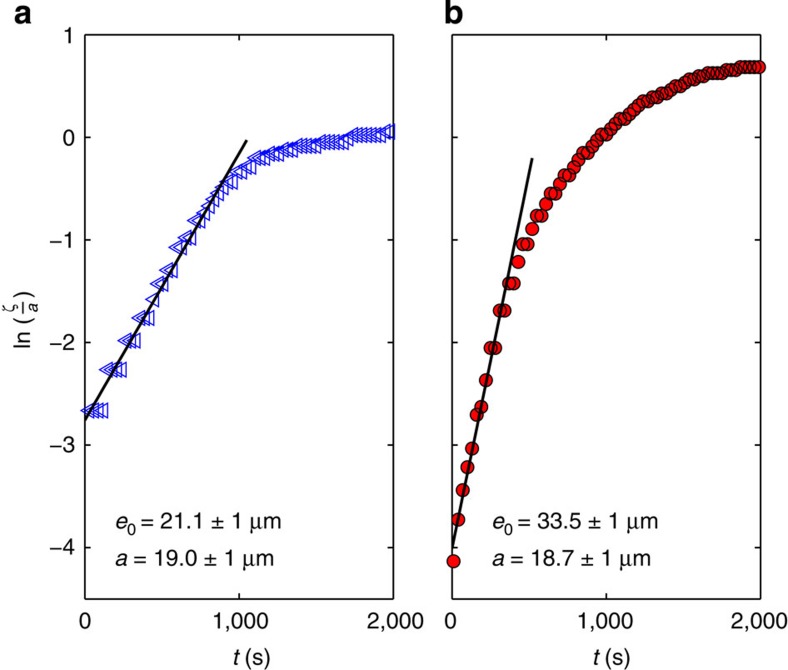
Temporal growth of the perturbation. Semi-logarithmic plot of the evolution of the perturbation on (**a**) a no-slip fibre (glass) and (**b**) a slip fibre (glass coated with AF2400). The radius of the fibre *a* and initial thickness of the polymer film *e*_0_ (see Fig. 1a) are indicated. The solid line is the best linear fit in the initial regime.

We now turn from the spatial morphology of the instability to the temporal evolution. From the experimental images (see Fig. 1b), we extract the maximal radius of an individual bulge as it develops, to obtain the amplitude *ζ* as a function of time. The linear stability analysis presented predicts a perturbation that grows exponentially with a dimensionless growth rate 1/τ* for the fastest growing mode (see equation (6)). Figure 3 displays typical data for the logarithm of the perturbation amplitude normalized by the radius of the fibre, *ζ*/*a*, as a function of *t*, for both no-slip and slip fibres. The data for both boundary conditions are consistent with the expected exponential growth in the early regime. Thus, the initial slopes of these curves provide reliable measurements of the growth rates.

**Figure 4:**
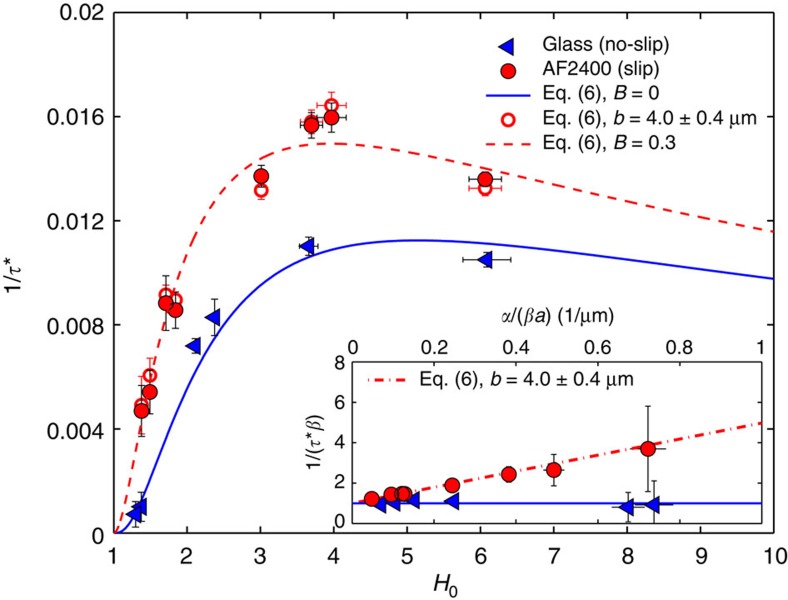
Influence of slip on the growth rate. The inset shows the dimensionless growth rate 1/τ* normalized by the no-slip case *β*, as a function of *α*/(*βa*), see equation (6) and equations (7a,b), 8. The slip length *b* is obtained from a best linear fit (dash-dotted) to the slip data. The error bars are calculated from the error in the geometry and the inaccuracy given by the growth rate measurement. The main curve shows the dimensionless growth rate of the fastest growing mode on no-slip (glass) and slip (AF2400) fibres, as a function of the dimensionless initial total radius *H*_0_=1+*e*_0_/*a* (see Fig. 1a). Open symbols represent growth rates calculated from equation (6) using *b*=4.0±0.4 μm and the respective experimental geometries. Also shown is the theoretical curve for no slip (equation (6), with *B*=0). Furthermore, the theoretical curve for slip (equation (6), with *B*=0.3) is plotted as a guide to the eye.

The dimensionless growth rates 1/τ* are shown in Fig. 4 for both slip and no-slip fibres as a function of the dimensionless initial total radius *H*_0_. We see that for both the slip and no-slip boundary conditions, the growth rates show a similar geometry dependence. The maxima for the slip and no-slip data can be easily understood: a decreasing growth rate as *H*_0_ converges to 1 is due to the diminishing thickness of the liquid film, and thus to the reduced mobility, whereas the decreasing growth rate for large *H*_0_ is due to smaller curvatures and thus a smaller driving force of the instability.

According to equation (6) with *B*=0 (no slip), a maximum in the growth rate is expected for *H*_0_=1+*e*_0_/*a*=5.15. This prediction is entirely consistent with the data. Thus, a coated fibre of a given diameter is maximally unstable when the ratio of the film thickness to the fibre radius is about *e*_0_/*a*∼4, consistent with an earlier theoretical study^5^. The capillary velocity is the only adjustable parameter in the no-slip case. We obtained *γ*/*η*=294±43 μm min^−1^, which is in agreement with previous data^33^ adjusted to the temperature used here, through the Wiliams–Landel–Ferry equation^37^,^38^. For the slip case, there are now two free parameters: the capillary velocity *γ*/*η* and the dimensionless slip length *B*. If we take the value of the capillary velocity to be that of the no-slip case, we are left with only one true fitting parameter.

To quantify the slip length in our system, the growth rates normalized by *β* are plotted as a function of *α*/(*βa*) (see inset of Fig. 4, equation (6) and equations (7a,b), 8). As expected, the no-slip data is consistent with the theoretical prediction of 1/τ*=*β* for all geometries. In contrast, the ratio 1/(τ^*^β) reveals that the amplification due to slip on the rise of the instability is more pronounced as *α*/(*βa*) increases. For the fibre radii used here, large *α*/(*βa*) corresponds to small *H*_0_ (see equations (7a,b), 8). The stronger influence of slip observed for smaller values of *H*_0_ is due to a non-zero velocity of polymer molecules at the solid–liquid interface^31^,^34^. For the smallest value of *H*_0_, corresponding to *e*_0_/*a*∼0.4, the slip-induced amplification factor of the growth rate is as large as ∼4. On the contrary, in thick polymer films, the impact of slip is diminished. From a best linear fit to the slip data shown in the inset of Fig. 4, the slip length is found to be *b*=4.0±0.4 μm. Obtaining a slip length in the range of micrometres is in accordance with former studies on the dewetting of entangled polymer films from substrates with a fluoropolymer coating^34^.

Having determined the value of *b* and knowing the fibre radii, we obtain *B* for each of the experimental geometries. Based on the theoretical model (equation (6)) corresponding growth rates can be calculated and are shown to be in excellent agreement with the experimental data (see Fig. 4). The dimensionless slip length *B*=*b*/*a* ranges from 0.25 to 0.37 in our experiments. To guide the eye, a typical curve calculated with *B*=0.3 is shown in Fig. 4. We see that for the fibres with the slip boundary condition, the growth rate is larger than in the no-slip case for a given geometry and the maximum of the growth rate 1/τ* corresponds to a smaller value of *H*_0_. As expected, a slippery surface facilitates a higher mobility and hence a faster growth of the instability. This enhanced mass transport also explains the horizontal shift of the maximum of the growth rate: for a given geometry—and thus curvature—the mobility of the slip case is increased in comparison with no-slip case. Therefore, the maximum of the growth rate is shifted to lower values of *H*_0_.

## Discussion

We report on the PRI of a viscous liquid PS layer on a solid fibre of radius *a*, when the boundary condition at the solid–liquid interface is varied between the classical case of no-slip and the relevant situation of slippage. The wavelength of the fastest growing mode on a slip fibre shows a linear dependence on the initial total polymer-fibre radius, *h*_0_=*a*+*e*_0_, and is not affected by the boundary condition, consistent with the lubrication theory developed. For both slip and no-slip fibres, we observe an exponential temporal growth of the instability at short times and the respective growth rates show a qualitatively similar geometry dependence. In the case of a slip fibre, the geometry corresponding to the maximum of the growth rate 1/τ* is shifted to a smaller value of *h*_0_/*a* and the rise of the instability is faster due to the added mobility at the solid–liquid interface. The slip-induced amplification of the growth rate is significant in a parameter range that is of paramount technological relevance. The linear stability analysis of the thin-film equation developed here is in excellent agreement with the data, valid for all Newtonian liquids, and provides a robust measure of two fundamental quantities: the capillary velocity *γ*/*η* and the slip length *b*.

## Methods

### Preparation of fibres

Glass fibres were prepared by pulling heated glass capillary tubes to final radius in the range 10<*a*<25 μm using a pipette puller (Narishige, PN30). To prepare the slip boundary condition, the glass fibres were hydrophobized by dip coating in a 0.5 wt% solution of AF2400 (Poly[4,5-difluoro-2,2-bis(trifluoromethyl)-1,3-dioxole-co-tetrafluoroethylene]) (Aldrich) in a perfluoro-compound solvent (FC72™, Fisher Scientific), to form an amorphous fluoropolymer layer (*T*_g_∼240 °C). A dip-coating speed of 1 mm s^−1^ resulted in an AF2400 layer with a thickness of 14±1 nm. The hydrophobized fibres were annealed in a vacuum chamber at 80 °C for 90 min to remove excess solvent.

### Preparation of homogeneous PS films

To prepare homogeneous PS films, a concentrated solution (35 wt%) of atactic PS (Polymer Source Inc.) with a molecular weight of 78 kg mol^−1^ and low polydispersity (*M*_w_/*M*_n_=1.05) was dissolved in chloroform (Fisher Scientific). A droplet of the highly viscous polymer solution was placed between two glass slides, forming a meniscus at the edge of the glass slides. We note that the chloroform does not dissolve the underlying AF2400 coating on the slip fibres. A slip or no-slip fibre could then be placed in the middle of the gap between the slides and pulled out of the droplet with a constant speed using a motorized linear translation stage. By varying the pulling speed *v*_0_ in the range 80<*v*_0_<150 mm s^−1^, we obtained film thicknesses that ranged from *e*_0_=5 to 93 μm after the solvent had evaporated.

### Experimental setup

The as-prepared samples were placed into a heated sample cell to initiate the PRI. With two ∼0.5-mm-thick spacers, the coated fibres were suspended above a reflective Si wafer (to improve contrast) and placed on a microscope hot stage (Linkam). A metal ring in direct contact with the hot stage supported a glass cover over the sample and Si wafer (see Supplementary Fig. 1 and Supplementary Methods), thereby ensuring good thermal contact and temperature control to within 1 °C. The surface profiles were analysed from the optical micrographs taken at various times using a custom-made edge detection software written in MATLAB.

## Additional information

**How to cite this article:** Haefner, S. *et al*. Influence of slip on the Plateau–Rayleigh instability on a fibre. *Nat. Commun*. 6:7409 doi: 10.1038/ncomms8409 (2015).

## Supplementary Material

Supplementary Information Supplementary Figure 1 and Supplementary Methods
