# Supplementary material for: Influence of slip on the Plateau–Rayleigh instability on a fibre
Source: Nat Commun. 2015 Jun 12;6:7409. doi: 10.1038/ncomms8409 (PMC4490368; doi:10.1038/ncomms8409)
Supplement: Supplementary Information — Supplementary Figure 1 and Supplementary Methods [file ncomms8409-s1.pdf]

Supplementary Figures

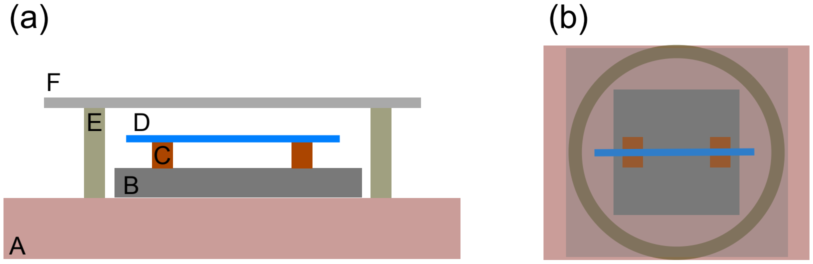

Supplementary Figure 1: **Schematic of the sample chamber.** Representation of (a) the side view and (b) the top view of the experimental setup. On top of a hot stage (A), a Si wafer (B) with two teflon spacers (C) is placed. Freely suspended filaments are realized by depositing the fibre (D) across the spacers. The fibre is surrounded by a metal ring (E) with a glass cover (F).

Supplementary Method

Fibre thin film equation with slip:

Here, we provide the main steps of the derivation of the fibre thin film equation (see Eqs. (2) and (3) in the article). Let us consider a viscous liquid film coating a cylindrical fibre of constant radius a (see Fig. 1 in the article). The axis of the fibre, along the x direction, is chosen as a reference for the radial coordinate r. We assume cylindrical invariance of the free-surface profile described by $r = h(x,t)$, at position x and time t. Furthermore, we assume an incompressible flow of the viscous Newtonian fluid, where gravity, disjoining pressure and inertia are negligible. Defining the local excess pressure $p(r,x,t)$ and the velocity field $\boldsymbol{v}(r,x,t)$, and assuming the lubrication approximation to first order, namely that the slopes of the profiles remain small and the velocity is mainly oriented along the fibre axis, $\boldsymbol{v} = v\boldsymbol{e}_x$, leads to the two projections of the Stokes equation:

$$\partial_x p \ = \ \eta\left(\partial_{rr}v + \frac{\partial_r v}{r}\right) \tag{1a}$$

$$\partial_r p \ = \ 0 \ , \tag{1b}$$

where η is the dynamical shear viscosity that we assume to be homogeneous and constant. The lubrication approximation is valid since the profile slopes remain small in comparison to 1 at early times – the regime in which the linear analysis is performed – and since the typical horizontal length scale of the flow is expected to be larger than the minimal wavelength of the Plateau-Rayleigh instability, that is given by $\lambda_{\text{min}} = 2\pi h_0 \gg e_0$. This is indeed always the case as even in the worst case geometry, $e_0 \approx 5a$, one has $e_0/\lambda_{\text{min}} \approx 0.13 \ll 1$. According to Supplementary Eq. (1b), the excess pressure is invariant in the radial direction, to first order in the lubrication approximation. Thus, the excess pressure is set by the Laplace boundary condition at the free surface which, within the small slope approximation, reads:

$$p \ = \ \gamma\left(\frac{1}{h} - h''\right) \ , \tag{2}$$

where γ denotes the air-liquid surface tension, taken to be homogeneous and constant, and where the prime denotes the derivative with respect to x. The two terms on the right hand side of Supplementary Eq. (2) correspond to the radial and axial curvatures of the free surface. Note that, as already mentioned in the body of the article, we have kept a second order lubrication term in the pressure contribution: the axial curvature $-h''$. This is because it is the lowest order term counterbalancing the driving radial curvature $1/h$, and it is thus crucial to obtain the actual threshold of the instability [9]. Regarding the boundary conditions, we assume no shear at the free surface and the Navier slip condition at the solid-liquid interface, namely:

$$\partial_r v|_{r=h} \ = \ 0 \tag{3a}$$

$$\partial_r v|_{r=a} \ = \ \frac{v|_{r=a}}{b} \ , \tag{3b}$$

where b denotes the slip length. Integrating Supplementary Eq. (1), together with Supplementary Eqs. (2) and (3), yields:

$$v \ = \ \frac{\gamma\left(h' + h^2 h'''\right)}{4\eta h^2}\left[2h^2\log\left(\frac{r}{a}\right) - r^2 + \frac{2b}{a}h^2 + a^2 - 2ab\right] \ , \tag{4}$$

for all $r \in [a,h]$. Volume conservation requires that:

$$\partial_t h + \frac{Q'}{h} \ = \ 0 \ , \tag{5}$$

where we introduced the volume flux per radian:

$$Q \ = \ \int_a^h \mathrm{d}r\, rv \ . \tag{6}$$

Combining Supplementary Eq. (5) together with Supplementary Eqs. (4) and (6) yields the fibre thin film equation:

$$\partial_t h + \frac{\gamma}{16\eta h}\left[\left(h' + h^2 h'''\right)\left(4h^2 \log\left(\frac{h}{a}\right) + \left(\frac{4b}{a} - 3\right)h^2 + 4a^2 - 8ab + \left(4ab - a^2\right)\frac{a^2}{h^2}\right)\right]' = 0 . \tag{7}$$

Finally, introducing the dimensionless variables (see Eq. (1) in the article) into Supplementary Eq. (7) yields the dimensionless fibre thin film equation (see Eqs. (2) and (3) in the article).

Experimental setup and details:

The as-prepared samples were annealed in ambient atmosphere at 180 °C - well above T_g - which causes the polystyrene films to melt and the PRI to develop. The evolution of the PRI was recorded with an inverted optical microscope (Olympus BX51). At various times, images were taken using a 5x magnification objective and a camera with a resolution of 1392 x 1040 pixels (QImaging, QIClick), resulting in a pixel size of 1.28 μm. To ensure free standing filaments, the coated fibres (D) were placed along two $\sim$ 0.5 mm thick teflon spacers (C) located on a Si wafer (B) (see Supplementary Figure 1). The Si wafer was used to improve the optical contrast. A metal ring (E) in direct contact with the hot stage supported a glass cover (F) over the sample and Si wafer. The temperature within the sample cell is uniform (within 1°C) and convection does not affect the measurements. By using spacers (C) made of teflon, we ensured that there is no drainage of the liquid PS film towards the edges (PS does not wet teflon). The surface profiles were analysed from the optical micrographs taken at various times, using a custom-made edge detection software written in MATLAB.
